# Supplementary material for: Reliability of CMV-IgG kinetics in the diagnosis of CMV primary infection: sensitivity, specificity, and clinical implications
Source: Microbiol Spectr. 2025 Jun 17;13(8):e00455-25. doi: 10.1128/spectrum.00455-25 (PMC12323311; doi:10.1128/spectrum.00455-25)
Supplement: Table S1 — Inter-assay variability in CMV-IgG titers: data from the external quality control program (2015-2022). [file spectrum.00455-25-s0001.docx]

|  |  |  |  |  |  |  |  |  |  |  |  |  |  |  |  |  |  |  |  |  |  |  |  |  |  |  |  |  |  |  |  |  |  |  |
| --- | --- | --- | --- | --- | --- | --- | --- | --- | --- | --- | --- | --- | --- | --- | --- | --- | --- | --- | --- | --- | --- | --- | --- | --- | --- | --- | --- | --- | --- | --- | --- | --- | --- | --- |
|  | **EQA sample name** | **LIAISON CMV IgG II** | | | | **IMMULITE 2000 CMV IgG** | | | | **Elecsys CMV IgG - Cobas e411, e601, e602** | | | | **Architect CMV IgG** | | | | **Alinity i CMV IgG** | | | | **VIDAS CMV IgG** | | | | **Access CMV IgG Cytomegalovirus Antibody** | | | | **Total of labs participating to the EQA** |  | **Higher titer on survey** | **Lowest titer on survey** | **Ratio: assay with the highest value to assay with the lowest value** |
|  |  | **Nb Labs** | **Mean** | **SD** | **CV (%)** | **Nb Labs** | **Mean** | **SD** | **CV (%)** | **Nb Labs** | **Mean** | **SD** | **CV (%)** | **Nb Labs** | **Mean** | **SD** | **CV (%)** | **Nb Labs** | **Mean** | **SD** | **CV (%)** | **Nb Labs** | **Mean** | **SD** | **CV (%)** | **Nb Labs** | **Mean** | **SD** | **CV (%)** |  |  | **Max** | **Min** |  |
|  | **2015-1** | 13 | 144,4 | 21,0 | 14,0 | 5 | 13,5 | 0,9 | 6,7 | 43 | 2409,4 | 130,0 | 5,4 | 25 | 392,4 | 23,0 | 5,8 |  |  |  |  | 40 | 107,2 | 17,0 | 16,0 | 20 | 343,5 | 36,0 | 10,0 | 141 |  | 2409,4 | 13,5 | 178,5 |
|  | **2015-2** | 15 | 118,8 | 9,6 | 8,1 | 6 | 12,6 | 0,8 | 6,3 | 44 | 96,108 | 8 | 8,3 | 26 | 182,2 | 20,0 | 11,0 |  |  |  |  | 39 | 68,2 | 10,0 | 15,0 | 22 | 379,6 | 33,0 | 8,7 | 146 |  | 379,6 | 12,6 | 30,0 |
|  | **2015-3** | 12 | 51,7 | 4,5 | 8,7 | 8 | 8,7 | 0,5 | 5,9 | 38 | 15,3 | 0,7 | 4,6 | 23 | 75,3 | 12,0 | 16,0 |  |  |  |  | 40 | 27,7 | 2,4 | 8,7 | 21 | 165,2 | 19,0 | 11,0 | 134 |  | 165,2 | 8,7 | 19,0 |
|  | **2016-2** | 12 | 85,7 | 9,2 | 11,0 | 7 | 9,1 | 0,9 | 10,0 | 37 | 154,7 | 3,6 | 2,3 | 25 | 208,0 | 15,0 | 7,0 |  |  |  |  | 40 | 51,5 | 4,8 | 9,3 | 23 | 549,5 | 90 | 16 | 137 |  | 549,5 | 9,1 | 60,5 |
|  | **2016-3** | 13 | 133,0 | 12,0 | 9,3 | 7 | 19,1 | 0,5 | 2,8 | 39 | 828,4 | 81,0 | 9,7 | 25 | 253,1 | 4,1 | 1,6 |  |  |  |  | 39 | 152.48 | 34 | 22 | 20 | 467,7 | 26 | 6 | 136 |  | 828,4 | 19,1 | 43,4 |
|  | **2016-4** | 13 | 35,4 | 6,8 | 19,0 | 7 | 8,8 | 0,4 | 4,8 | 38 | 3,9 | 0,3 | 6,4 | 24 | 63,0 | 9,6 | 15,0 |  |  |  |  | 37 | 20,5 | 3,3 | 16,0 | 24 | 221,2 | 29,0 | 13,0 | 136 |  | 221,2 | 3,9 | 56,8 |
|  | **2017-1** | 13 | 127,3 | 7,0 | 5,5 | 8 | 13,6 | 0,5 | 3,9 | 32 | 2516,4 | 130,0 | 5,2 | 27 | 343.89 | 110 | 32 |  |  |  |  | 32 | 104,9 | 14,0 | 13,0 | 24 | 361,9 | 41,0 | 11,0 | 128 |  | 2516,4 | 13,6 | **184,7** |
|  | **2017-2** | 14 | 105,1 | 3,8 | 3,6 | 8 | 12,4 | 1,1 | 8,9 | 32 | 73,2 | 2,7 | 3,6 | 29 | 200,2 | 16,0 | 8,1 |  |  |  |  | 32 | 71,6 | 6,0 | 8,4 | 24 | 406,9 | 31,0 | 7,5 | 131 |  | 406,9 | 12,4 | 32,8 |
|  | **2017-4** | 15 | 52,5 | 4,4 | 8,4 | 7 | 8,8 | 0,4 | 4,1 | 35 | 15,3 | 0,6 | 4,1 | 27 | 84,6 | 9,6 | 11,0 |  |  |  |  | 31 | 24,4 | 1,6 | 6,7 | 23 | 173,7 | 19,0 | 11,0 | 131 |  | 173,7 | 8,8 | 19,7 |
|  | **2018-2** | 13 | 90,0 | 14,0 | 15,0 | 5 | 13,9 | 1,3 | 9,1 | 29 | 366,8 | 8,7 | 2,4 | 24 | 233,9 | 14,0 | 5,9 | 4 | 353,6 | 95,0 | 27,0 | 31 | 44,1 | 7,7 | 17,0 | 24 | 420,3 | 50,0 | 12,0 | 121 |  | 420,3 | 13,9 | 30,2 |
|  | **2018-4** | 13 | 19,1 | 2,3 | 12,0 | 5 | 2,1 | 0,1 | 5,9 | 28 | 2,8 | 0,1 | 4,0 | 26 | 34,3 | 6,4 | 19,0 | 4 | 27,0 | 1,0 | 3,9 | 24 | 6,0 | 0,0 | 0,0 | 3 | 16.087 | 0.98 | 6.1 | 94 |  | 34,3 | 2,1 | 16,2 |
|  | **2019-1** | 21 | 53,2 | 6,6 | 12,0 | 6 | 8,9 | 0,8 | 9,2 | 40 | 16,2 | 0,6 | 3,9 | 33 | 92,8 | 7,2 | 7,8 | 5 | 101,4 | 2,6 | 2,6 | 30 | 27,0 | 2,7 | 10,0 | 18 | 70,0 | 9,6 | 14,0 | 142 |  | 101,4 | 8,9 | 11,4 |
|  | **2019-2** | 21 | 135,9 | 14,0 | 10,0 | 5 | 15,3 | 1,2 | 7,8 | 42 | 2339,4 | 230,0 | 9,7 | 31 | 357.67 | 82 | 23 | 8 | 372,3 | 37,0 | 9,8 | 29 | 93,9 | 8,0 | 8,5 | 15 | 310,3 | 38,0 | 12,0 | 138 |  | 2339,4 | 15,3 | 153,3 |
|  | **2019-4** | 21 | 103,9 | 6,1 | 5,9 | 5 | 10,9 | 0,3 | 3,1 | 41 | 430,7 | 28,0 | 6,5 | 29 | 174,7 | 12,0 | 6,8 | 12 | 166,0 | 3,6 | 2,2 | 28 | 60,9 | 6,8 | 11,0 | 12 | 242,7 | 49,0 | 20,0 | 131 |  | 430,7 | 10,9 | 39,4 |
|  | **2020-1** | 23 | 21,1 | 1,7 | 8,1 | 3 | 2,2 | 0,1 | 3,3 | 50 | 2,8 | 0,1 | 4,7 | 30 | 33,8 | 4,3 | 13,0 | 13 | 36,0 | 2,4 | 6,7 | 10 | 6,0 | 0,0 | 0,0 | 10 | 16,6 | 0,7 | 4,4 | 123 |  | 36,0 | 2,8 | 12,8 |
|  | **2020-3** | 24 | 22,8 | 3,0 | 13,0 | 3 | 3,8 | 0,4 | 9,8 | 55 | 163,3 | 14,0 | 8,8 | 26 | 31,3 | 2,3 | 7,4 | 15 | 31,6 | 1,7 | 5,5 | 25 | 13,0 | 0,0 | 0,0 | 17 | 53,0 | 3,0 | 5,7 | 147 |  | 163,3 | 13,0 | 12,6 |
|  | **2020-4** | 24 | 84,1 | 5,4 | 6,4 | 3 | 8,4 | 1,2 | 14,0 | 54 | 68,2 | 8,8 | 13,0 | 25 | 109,6 | 10,0 | 9,5 | 15 | 123,9 | 9,2 | 7,4 | 26 | 40,9 | 4,8 | 12,0 | 16 | 49,5 | 6,7 | 14,0 | 145 |  | 123,9 | 40,9 | 3,0 |
|  | **2021-3** | 25 | 59,2 | 4,8 | 8,1 | 4 | 8,3 | 0,3 | 3,6 | 53 | 1,6 | 0,1 | 9,0 | 21 | 47,6 | 2,9 | 6,2 | 18 | 46,6 | 2,3 | 5,0 | 27 | 17,5 | 1,3 | 7,3 | 20 | 52,9 | 5,6 | 11,0 | 146 |  | 59,2 | 1,6 | 38,1 |
|  | **2021-4** | 26 | 141,4 | 14,0 | 9,6 | 4 | 18,9 | 1,0 | 5,3 | 18 | 2120,9 | 120,0 | 5,7 | 22 | 522.74 | 190 | 36 | 17 | 519.98 | 160 | 31 | 29 | 73,7 | 8,8 | 12,0 | 19 | 442,5 | 51 | 11 | 114 |  | 2215,2 | 73,7 | 30,0 |
|  | **2022-1** | 27 | 138,9 | 9,5 | 6,8 | 3 | 25,03 | 1,8 | 7,0 | 21 | 2674,4 | 130,0 | 4,7 | 23 | 527,5 | 42,0 | 8,0 | 23 | 529,2 | 29,0 | 5,6 | 25 | 96,7 | 19,0 | 20,0 | 16 | 446,8 | 52 | 12 | 112 |  | 2837,7 | 96,7 | 29,3 |
|  | **2022-4** | 26 | 132,2 | 7,5 | 5,7 | 3 | 21,5 | 1,6 | 7,6 | 15 | 503,0 | 22,0 | 4,4 | 23 | 236,4 | 13,0 | 5,4 | 24 | 258,9 | 32,0 | 12,0 | 23 | 106,7 | 14,0 | 13,0 | 14 | 414,4 | 43,0 | 10,0 | 101 |  | 503,0 | 106,7 | 4,7 |

**Supplementary Table S1: Inter-Assay Variability in CMV-IgG Titers: Data from the External Quality Control Program (2015-2022)**

This table shows the variability of CMV-IgG titers measured using different commercial immunoassays as part of an External Quality Assessment (EQA) program.

For each sample, the number of laboratories using each assay (Nb Labs), the mean CMV-IgG titer, the standard deviation (SD), and the coefficient of variation (CV%) are reported.

The data illustrate significant discrepancies between assays, highlighting the impact of assay selection on CMV-IgG quantification and the potential implications for clinical interpretation.

Only assays used at least by five participants were included for calculations in this study (excluded data in grey-colored cells). Reagents CV > 20% were excluded for the analysis (orange-colored cells).

Units: U/mL (Cobas), AU/mL (Architect, Alinity, Access, Vidas, Liaison), and S/CO (Immulite).
